# Exploring protein symmetry at the RCSB Protein Data Bank

**DOI:** 10.1042/ETLS20210267

**Published:** 2022-07-08

**Authors:** Jose M. Duarte, Shuchismita Dutta, David S. Goodsell, Stephen K. Burley

**Affiliations:** 1 Research Collaboratory for Structural Bioinformatics Protein Data Bank, San Diego Supercomputer Center, University of California, San Diego, La Jolla, CA 92093, U.S.A.; 2 Research Collaboratory for Structural Bioinformatics Protein Data Bank, Rutgers, The State University of New Jersey, Piscataway, NJ 08854, U.S.A.; 3 Institute for Quantitative Biomedicine, Rutgers, The State University of New Jersey, Piscataway, NJ 08854, U.S.A.; 4 Rutgers Cancer Institute of New Jersey, Rutgers, The State University of New Jersey, New Brunswick, NJ 08903, U.S.A.; 5 The Scripps Research Institute, La Jolla, CA 92037, U.S.A.; 6 Department of Chemistry and Chemical Biology, Rutgers, The State University of New Jersey, Piscataway, NJ 08854, U.S.A.

**Keywords:** biomolecular symmetry, Protein Data Bank, protein structure

## Abstract

The symmetry of biological molecules has fascinated structural biologists ever since the structure of hemoglobin was determined. The Protein Data Bank (PDB) archive is the central global archive of three-dimensional (3D), atomic-level structures of biomolecules, providing open access to the results of structural biology research with no limitations on usage. Roughly 40% of the structures in the archive exhibit some type of symmetry, including formal global symmetry, local symmetry, or pseudosymmetry. The Research Collaboratory for Structural Bioinformatics (RCSB) Protein Data Bank (founding member of the Worldwide Protein Data Bank partnership that jointly manages, curates, and disseminates the archive) provides a variety of tools to assist users interested in exploring the symmetry of biological macromolecules. These tools include multiple modalities for searching and browsing the archive, turnkey methods for biomolecular visualization, documentation, and outreach materials for exploring functional biomolecular symmetry.

## Functional symmetry of proteins

**Figure 1.**
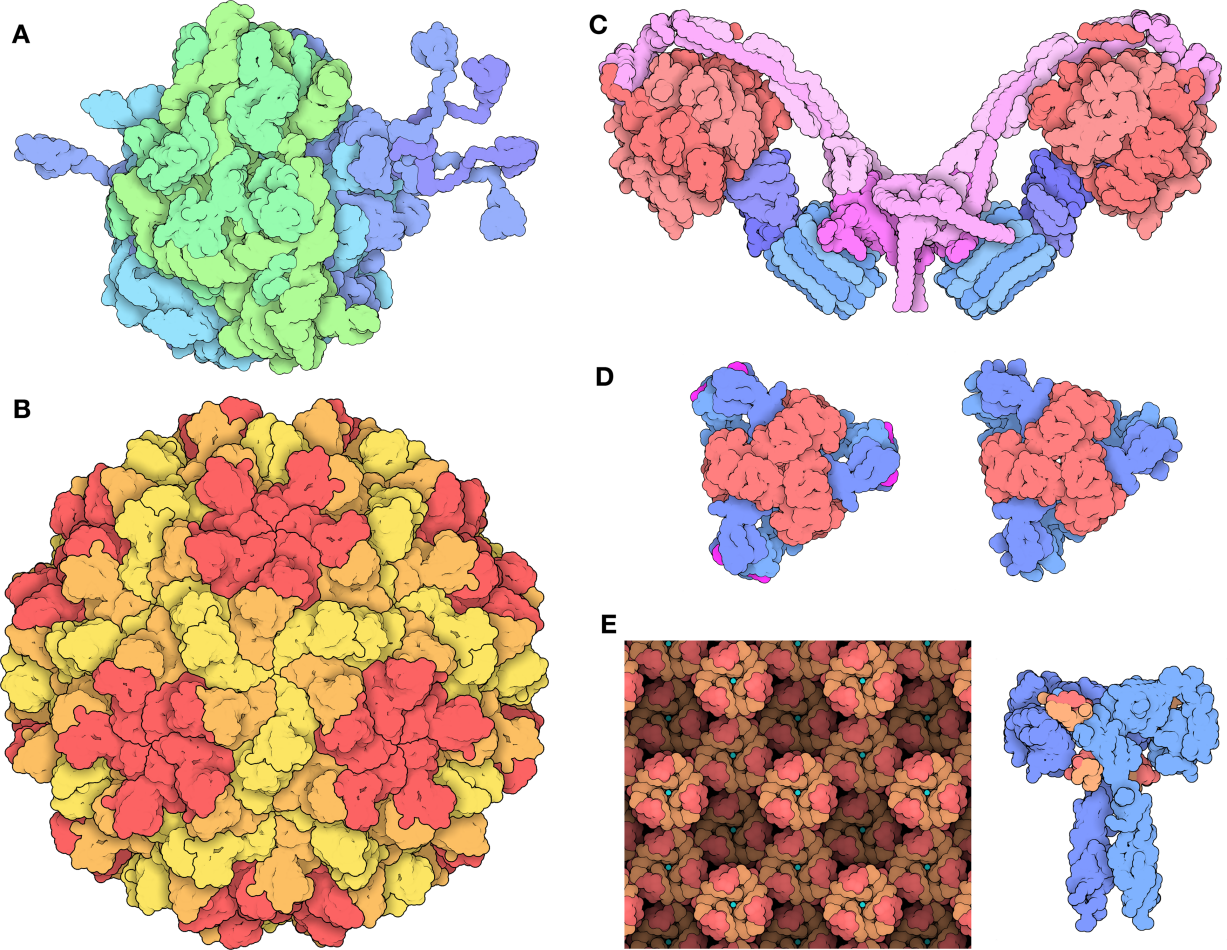
Examples of functional symmetry. (**A**) Ribosomes are among the largest asymmetric assemblies found in living organisms (based on PDB ids 4v4q, 1rqu [39,40]). (**B**) Virus capsids use icosahedral quasisymmetry to build large structures from multiple identical subunits packed into slightly different local environments (PDB id 2tbv [41]). (**C**) ATP synthase has a chemical F1 motor with three-fold symmetry (red) and a membrane-embedded F0 motor with ten-fold symmetry (turquoise) connected by an asymmetric axle (dark blue), which are then arranged in pairs with 2-fold symmetry (PDB id 6b8h [14]). (**D**) Aspartate carbamoyltransferase is a symmetrical allosteric enzyme that transitions between inactive (left) (PDB id 5at1 [42]) and active (right) (PDB id 1d09 [43]) conformations. (**E**) Insulin is stored in pancreatic cells as micro-crystals of hexamers of heterodimers stabilized by zinc ions (left, red and tan denoting insulin α and β chains, respectively; cyan circle: zinc ion) (PDB id 4ins [44]), but is active as a single heterodimer when bound to its receptor (receptor in blue at right) (PDB id 6pxv [45]). Images adapted from Molecule of the Month [46] and rendered here at a consistent scale.

Symmetry of biological macromolecules is a classic example of the structural biology tenet: *function follows form*. When browsing the PDB archive, we find myriad examples of individual proteins arranged in the shape of rings, containers, channels, filaments, sheets, and complex molecular machines, all tailored to fulfill particular functional roles. Figure 1 exemplifies the scope of what is already known about symmetric assemblies. In most cases, these assemblies are composed of multiple identical subunits arranged symmetrically. Such arrangements were predicted from first principles before any atomic-level three-dimensional (3D) structures of biomolecules were determined. In 1956, for example, Crick and Watson correctly predicted that cubic symmetries would be uniquely suited to building the hollow shells of spherical viruses [1]. Principles of biomolecular symmetry, its functional and evolutionary consequences, and the many structural and functional exceptions to symmetry have been extensively covered elsewhere [2–9], and are beyond the scope of this brief review. After a short introduction, we will devote the bulk of this article to describing tools at the Research Collaboratory for Structural Biology (RCSB) Protein Data Bank (PDB) for finding, visualizing, analyzing, and exploring aspects of symmetry within the PDB archive of more than 190 000 experimentally determined, atomic-level 3D structures of biological macromolecules.

Monod succinctly proposed ‘*finiteness, stability, and self-assembly*’ as drivers for the evolution of symmetrical assemblies [10], and since then, the many morphological, energetic, and evolutionary advantages of symmetry have been extensively studied and confirmed [2–9]. Figure 1A,B exemplify one aspect of these imperatives: *genetic parsimony*. Large assemblies with finite size can be encoded and self-assembled using a small number of genes if they are built of subunits arranged with one or more intersecting rotational symmetries (i.e. point group symmetry). To demonstrate the potential problem, the ribosome is one of the largest asymmetric assemblies in the cell. This asymmetry is needed because it performs a complex, asymmetric biochemical function, moving directionally along a strand of mRNA and positioning multiple tRNA and protein factors to assemble the nascent protein chain. Cells make a huge investment in manufacturing ribosomes. For example, yeast ribosomes include ∼5500 nucleotides of RNA and 78 distinct proteins, and ∼200 accessory proteins are required to assemble them [11]. This expense is vast compared with many viruses, which make enormous capsids but cannot afford to encode a large number of proteins to package their genetic material. Instead, they build capsids using high degrees of symmetry and quasisymmetry (approximate icosahedral symmetry with multiples of 60 subunits) [12], while only committing modest genomic space to encode the subunit(s). A recent theoretical study supports the hypothesis that this parsimony may also be one of the driving forces for evolution of symmetric assemblies in cells [13].

Figure 1C shows a complex example of functional *symmetry breaking*. Deviations from perfect symmetry occur widely in nature when macromolecular assemblies must carry out specialized tasks. ATP synthase is a remarkable example. The yeast mitochondrial version includes two chemical motors [14]. The first motor (F1) is driven by ATP and has three-fold symmetry, with three binding-sites for ATP, but is pushed away from perfect symmetry by an asymmetric axle that threads through the center of the motor running along the cyclic symmetry axis. Progressive transition between three conformations of these three subunits ensures directional rotation of the motor. In addition, this motor shows six-fold pseudosymmetry, with three structurally similar subunits separating the three ATP-binding subunits. The second motor (F0) has ten-fold symmetry and interacts with an asymmetric motor subunit that drives the rotation of the cylindrical ring of subunits. In the cell, two of these assemblies are brought together to form an angled dimeric assembly that plays a role in modeling the shape of membranes within the mitochondrion [15].

Figure 1D exemplifies a major functional advantage of assemblies composed of multiple subunits: *cooperativity*. Allosteric enzymes are most often symmetric assemblies, and more specifically, they frequently have dihedral symmetry. Dihedral symmetries have several structurally-unique axes of rotational symmetry, forming multiple structurally-unique interfaces between subunits. It has been hypothesized that these different interfaces provide additional opportunities for the evolution of structural switches used in allosteric transitions [16]. Related to this, molecules such as antibodies and lectins use symmetrical assembly to bring together multiple copies of a subunit, allowing cooperative binding to adjacent sites on a target.

Translational symmetries in one, two, or three dimensions are also used to support specialized biochemical functions, particularly when large assemblies are needed. For example, insulin, itself an α/β-heterodimer, is stored in small three-dimensional crystals inside pancreatic secretory vesicles, which then dissociate into hexamers and then the active heterodimeric hormone when released into the bloodstream (Figure 1E) [17]. Cytoskeletal filaments and filamentous viruses often combine one-dimensional translation with a rotation yielding helical symmetry, as proposed by Pauling in 1953 [18]. Bacterial S-layers are examples of a two-dimensional translational lattice, used to coat the surface of a bacterial cell with a protective protein mesh resembling chainmail armor [19]. Translational symmetries are also an integral part of biomolecular structure determination by X-ray crystallography, which may cause methodological challenges, for example, when the helical symmetry of a biological filament does not conform to the allowed symmetry of possible crystal packing arrangements [20].

## The PDB archive and symmetry

The PDB is a core resource central to the global biodata ecosystem serving many millions of users drawn from diverse scientific and educational communities. It provides a permanent and expertly curated data archive [21–25] for structural biologists to disseminate their results, promotes reproducibility of the structural biology scientific literature, and makes biomolecular structure information freely available to a wide community of researchers, educators, students, and the general public without limitations on data usage. The PDB was established in 1971 at Brookhaven National Laboratory as the first open-access, digital-data resource in biology [26]. Since 2003, the PDB has been managed by the Worldwide Protein Data Bank partnership (wwPDB; wwPDB.org) [27,28]. Member organizations of the wwPDB (RCSB Protein Data Bank, RCSB PDB; Protein Data Bank in Europe, PDBe; Protein Data Bank Japan, PDBj; Electron Microscopy Data Bank, EMDB; and Biological Magnetic Resonance Bank, BMRB) together curate and annotate 3D biostructure data deposited by scientists from around the globe, and make it publicly, freely, and easily available through user-friendly web portals and host services. RCSB PDB, a founding member of the wwPDB, is responsible for US PDB operations, and serves as the wwPDB-designated PDB Archive Keeper. The RCSB PDB web portal (RCSB.org) supports millions of users worldwide [29–31]. In 2021, the website was visited each month by an average of ∼757 000 unique visitors according to Google Analytics, with ∼4.7 million unique visitors annually. A total of 257.71 TB of data were accessed. In 2021, 1.8 billion data files in various file formats, including structure files, experimental data files, chemical and molecular reference data files, and validation reports, were downloaded and/or viewed from RCSB PDB-hosted FTP and websites. Additional data were downloaded from wwPDB partners PDBe and PDBj for a total of 2.3 billion data files. This research-focused website provides tools and services that support users across scientific disciplines to access, analyze, and visualize up-to-date structural views of proteins and nucleic acids important to fundamental biology, biomedicine, and bioenergy sciences.

Symmetry is found at many levels in the PDB archive. At the methodological level, X-ray crystallography relies on an extensive body of knowledge about symmetries of crystals. A comprehensive set of *space group*s (standard combinations of allowable lattices with self-consistent rotations and translations) defines allowable packing arrangements of molecules within a lattice. The *asymmetric unit* is a key concept in this formalism, defining the unique repeated unit making up the crystal lattice. Typically, atomic coordinates for only the asymmetric unit are deposited into the PDB archive, since the entire lattice may be computationally generated using the geometric space group transformation matrices. A challenge emerges, however, when looking at symmetric biomolecules: the relevant biological state of an assembly does not always correspond to the crystallographic *asymmetric unit*. This challenge is further compounded for large assemblies, such as virus capsids, for which structural biologists often improve structure-determination methodology by imposing so-called non-crystallographic symmetry in cases where multiple identical subunits comprise the *asymmetric unit*. In such cases, the PDB structure may include only one of these subunits, together with the 3D transformation matrices required to generate the atomic coordinates for the remaining subunits.

**Figure 2.**
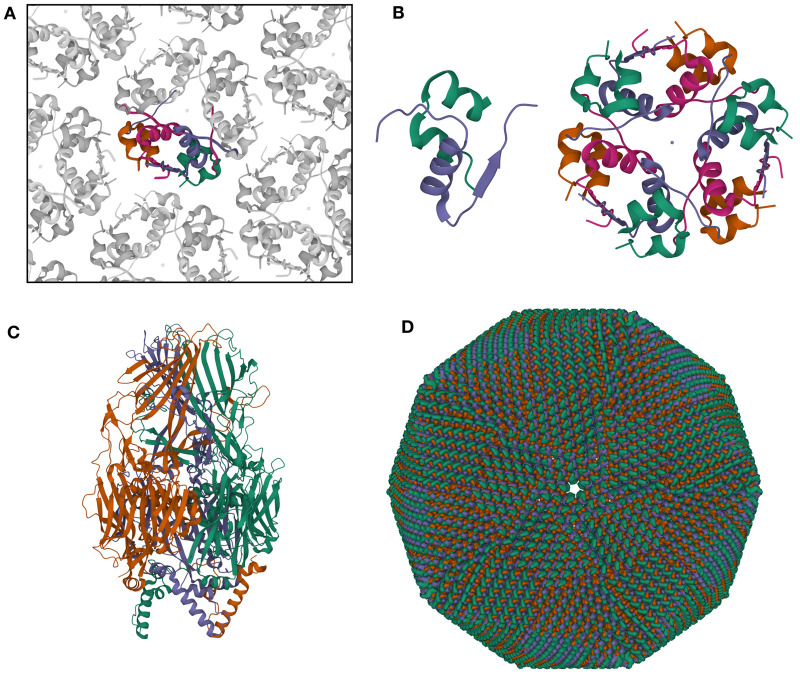
Examples of biological assemblies in PDB. (**A**) The hexagonal crystal lattice of insulin (also shown in Figure 1E) has two unique copies of the heterodimeric protein hormone comprising the asymmetric unit (colored), so the PDB structure includes atomic coordinates for two insulin molecules, corresponding to four protein chains. (**B**) Two biological assemblies may be produced from this lattice, choosing one of the two copies for the active homodimeric form (PDB id 4ins, biological assemblies 1 and 2), and the hexamer of heterodimers visible in the crystal lattice, which is the inactive storage form of the hormone (PDB id 4ins, biological assembly 3). (**C**) The PDB structure for faustovirus, determined by cryo-electron microscopy, includes coordinates for one trimeric protomer of the virus as the asymmetric unit. (**D**) Atomic coordinates for the entire capsid may be generated using the 2760 transformation matrices provided in PDB structure 5j7v [47]. Visualized with Mol*.

In practice, the vast majority of PDB users are not expert in crystallographic methods (estimated to be ∼99%), so RCSB.org provides files that include the presumed *biological assembly* for each structure, removing the need for non-expert users to generate the atomic coordinates (Figure 2). In some cases, the definition of this *biological assembly* may not be obvious, so two methods are used to ascertain the most likely arrangement of macromolecules constituting the assembly. PDB depositors are asked to define an ‘author assigned’ *biological assembly*, and this is presented as the preferred assembly on the RCSB.org website. Second, software (most often PISA [32]) is used to identify likely *biological assemblies* based on the size of interfaces between protomers and their estimated importance in terms of overall stability.

**Figure 3.**
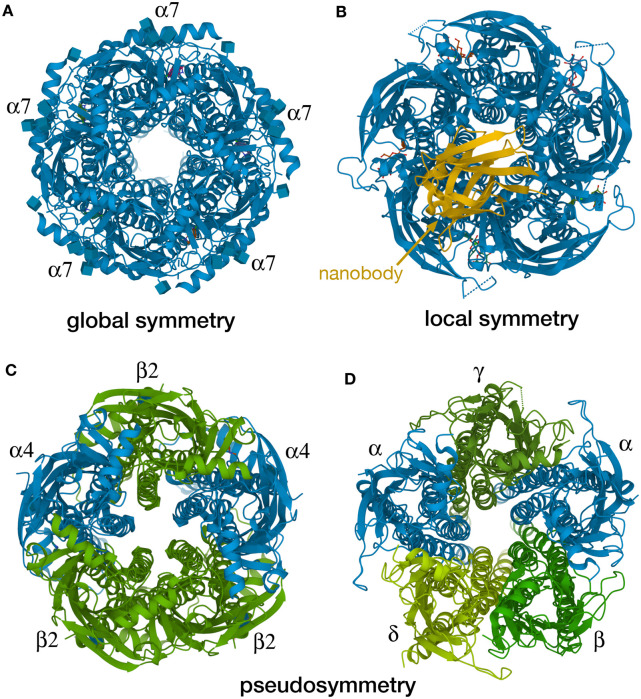
Types of symmetry annotated at RCSB PDB. Four examples of pentameric ligand-gated ion channels are shown here, viewed down the central pore. (**A**) The alpha7 nicotinic channel is composed of identical subunits with 5-fold rotational global symmetry. (**B**) The ELIC channel complex with a nanobody shows local 5-fold symmetry for the pentameric channel, but overall asymmetry in the entire complex. The alpha4beta2 nicotinic receptor (**C**) and the torpedo ray acetylcholine receptor (**D**) are pseudo-symmetric pentameric complexes, composed of several types of structurally-similar subunits with approximate 5-fold symmetry. Visualized in Mol* from PDB structures 7kox [48], 6ssp [49], 5kxi [50], 2bg9 [51].

It might appear at first glance that symmetry should be easy to define and evaluate, but in biology there are inevitable gray areas and exceptions. To address these challenges, the RCSB PDB currently evaluates three types of symmetry: global symmetry, local symmetry, and pseudosymmetry (Figure 3). *Global symmetry* is the most obvious and the most common: these are cases wherein the entire macromolecular assembly is defined by a single type of symmetry, such as point group or helical symmetry. For global symmetry calculations, individual components are considered equivalent when they are >95% sequence identical, which allows for analysis of macromolecular machines containing quasi-identical subunits. Complexes with *local symmetry* have portions that are symmetrical, but the overall symmetry is broken by association of subunits with different symmetry. Currently local symmetries are calculated for assemblies lacking global symmetry (i.e. when they are identified as C1). Assemblies with *pseudosymmetry* include two or more types of homologous subunits that form an assembly with approximate symmetry, if homologous subunits are considered to be equivalent. In this case, subunits are considered equivalent when constituents are more than 40% sequence identical or the α-carbon atoms of their structures align with root-mean-square-deviations (RMSDs) <3 Å. Detection of symmetry at RCSB PDB is performed by a custom algorithm that is implemented within the BioJava open-source software library [33]. The algorithm detects symmetry by efficiently superposing the subunits in a combinatorial fashion, finding rotation axes and orders. The algorithm runs as part of the RCSB PDB weekly update process, keeping the symmetry annotations up-to-date for the whole archive. To save computation time, the calculation is performed only for entries that are new or modified.

**Figure 4.**
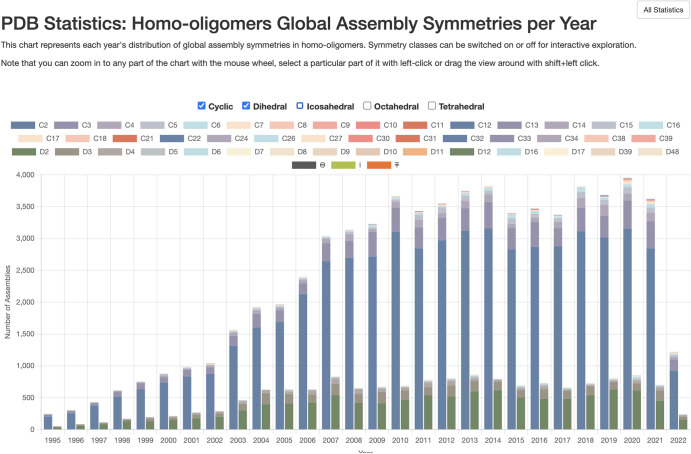
Symmetry statistics available at https://www.rcsb.org/stats/symmetry/growth. In this screenshot only dihedral and cyclic symmetries are shown, using the checkboxes near the top. The interactive view available on RCSB.org supports further exploration.

Table 1 provides a general survey of symmetries detected within current holdings for homo-oligomeric assemblies. Figure 4 presents the distribution of observed symmetries for structures deposited each year since the inception of the PDB. These include structure entries from all methods of structure determination, including structures from X-ray crystallography, NMR spectroscopy, and cryoelectron microscopy. Not surprisingly, X-ray crystallography has proven to be an amenable method for determination of symmetrical assemblies: 38% of crystallographic entries have some type of symmetry. Cryoelectron microscopy is similar, at 41%, however NMR has primarily been used to determine asymmetric, monomeric structures, with 10% of current entries showing some symmetry. Similar high-level statistics are available on the RCSB.org website at https://www.rcsb.org/stats/symmetry/growth to give users quick overviews of current archival content. RCSB.org also provides extensive annotations for all structures that facilitate deeper study by interested researchers. For example, a recent study of functional determinants of protein assembly [16] correlated homomeric symmetries with a variety of functional annotations, for example, finding a correlation between dihedral symmetries and metabolic enzymes. With the RCSB PDB Search Application Programming Interface (API), it is possible, for example, to programmatically query for the distribution of symmetry types and enzyme classification. A worked example is included on the RCSB PDB website at https://search.rcsb.org/#search-example-14, querying the distribution of enzyme classification terms per symmetry type for homo-oligomers. (N.B.: Identical searches, using the same API, can be made from the RCSB.org Advanced Search webpage.)

**Table 1:** Global symmetries for homo-oligomeric assemblies in current PDB holdings (as of April 20th 2022)

Class	Stoichiometry	Symmetry type	Count redundant^1^	Count non-redundant^2^
Cyclic	2	C2	56 568	13 590
Cyclic	3	C3	6836	1639
Cyclic	4	C4	1920	394
Cyclic	5	C5	1173	173
Cyclic	6	C6	699	204
Cyclic	7	C7	247	67
Cyclic	8	C8	95	35
Cyclic	9	C9	48	21
Cyclic	10	C10	28	13
Cyclic	11	C11	52	11
Cyclic	12	C12	61	26
Cyclic	13	C13	7	5
Cyclic	14	C14	16	9
Cyclic	15	C15	21	12
Cyclic	16	C16	7	2
Cyclic	17	C17	7	2
Cyclic	18	C18	2	1
Cyclic	21	C21	1	1
Cyclic	22	C22	3	2
Cyclic	24	C24	2	2
Cyclic	26	C26	1	1
Cyclic	27	C27	1	1
Cyclic	30	C30	1	1
Cyclic	31	C31	1	1
Cyclic	32	C32	1	1
Cyclic	33	C33	2	2
Cyclic	34	C34	3	1
Cyclic	38	C38	1	1
Cyclic	39	C39	2	1
Dihedral	4	D2	10 688	2319
Dihedral	6	D3	3036	908
Dihedral	8	D4	1030	318
Dihedral	10	D5	385	102
Dihedral	12	D6	219	70
Dihedral	14	D7	116	25
Dihedral	16	D8	55	23
Dihedral	18	D9	11	5
Dihedral	20	D10	4	3
Dihedral	22	D11	4	2
Dihedral	24	D12	2	2
Dihedral	32	D16	2	2
Dihedral	34	D17	4	2
Dihedral	78	D39	5	1
Dihedral	96	D48	1	1
Helical	n^3^	H	508	248
Icosahedral	60	I	483	179
Octahedral	24	O	608	69
Tetrahedral	12	T	473	145

1 ‘Redundant' where all PDB assemblies are counted;

2 ‘Non-redundant' where assemblies are clustered by 50% sequence identity;

3 Helical symmetries are unbounded and helices of arbitrary lengths may be generated.

## Tools for exploring protein symmetry at the RCSB PDB website

Given that symmetry is a pervasive property of PDB structures that is often essential for biological function, RCSB.org provides multiple methods for identifying and exploring symmetry. These tools fall into three general categories: at-a-glance annotation of symmetry and stoichiometry of each structure, symmetry-specific search and browse tools, and interactive 3D visualization of molecular symmetry.

The RCSB.org Structure Summary Page (SSP) for each PDB structure includes annotations related to symmetry. These annotations include symmetry types (cyclic, helical, icosahedral, etc.), symmetry classes for assemblies with global, local or pseudo-symmetric point groups, and stoichiometry of subunits in the assembly. Options are available to view 3D structures of these assemblies in Mol* [34] and display relevant symmetry axes. In addition, a link is provided to search for similar assemblies across the PDB archive. This tool performs a real-time search of all assemblies in the PDB archive, based on the BioZernike algorithm [35] that matches global shapes of assemblies, no matter their size. The method by which the assembly was defined (author-assigned or programmatic) is presented together with experimental evidence of the oligomerization state of the assembly (wherever possible).

**Figure 5.**
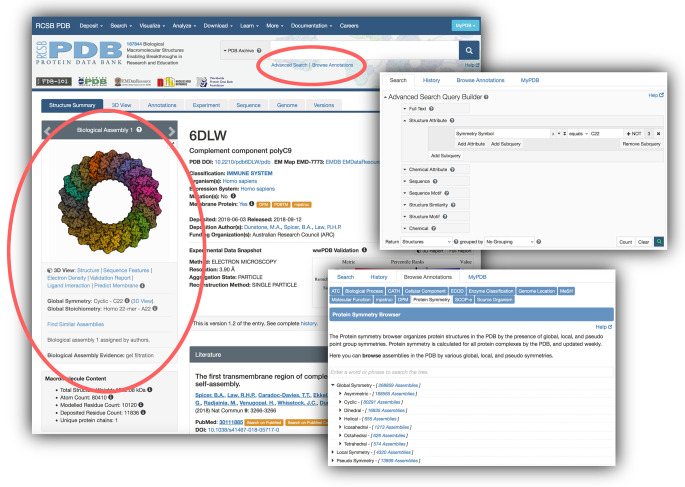
RCSB PDB tools for exploring symmetry. The RCSB.org Structure Summary Page for each PDB structure includes images of biological assemblies and asymmetric units and a summary of symmetries found within the assembly (circled at left). Two tools are provided to find proteins with particular symmetries (circled at top and insets at right): ‘Advanced Search’ queries the archive based on symmetry characteristics and ‘Browse Annotations: Protein Symmetry’ offers a drill-down tree browser of symmetry types.

Several tools are available for identifying macromolecular assemblies with particular symmetry (Figure 5). The RCSB.org Advanced Search page includes a wide range of searchable ‘Assembly Features’, including point group symmetry symbol, oligomeric state, symmetry type (cyclic, helical, *etc*.), and symmetry class (global, local, pseudo). These search attributes may be combined with other search functions available from the Advanced Search page (structural or chemical attributes, sequence, *etc*.) to develop more targeted searches. When search results are returned, a ‘Refinement’ option is provided in the left-hand menu that allows narrowing of any search result based on symmetry types and a variety of other annotated features. A Browse functionality is also available, providing direct links to all holdings with a particular symmetry symbol or class.

**Figure 6.**
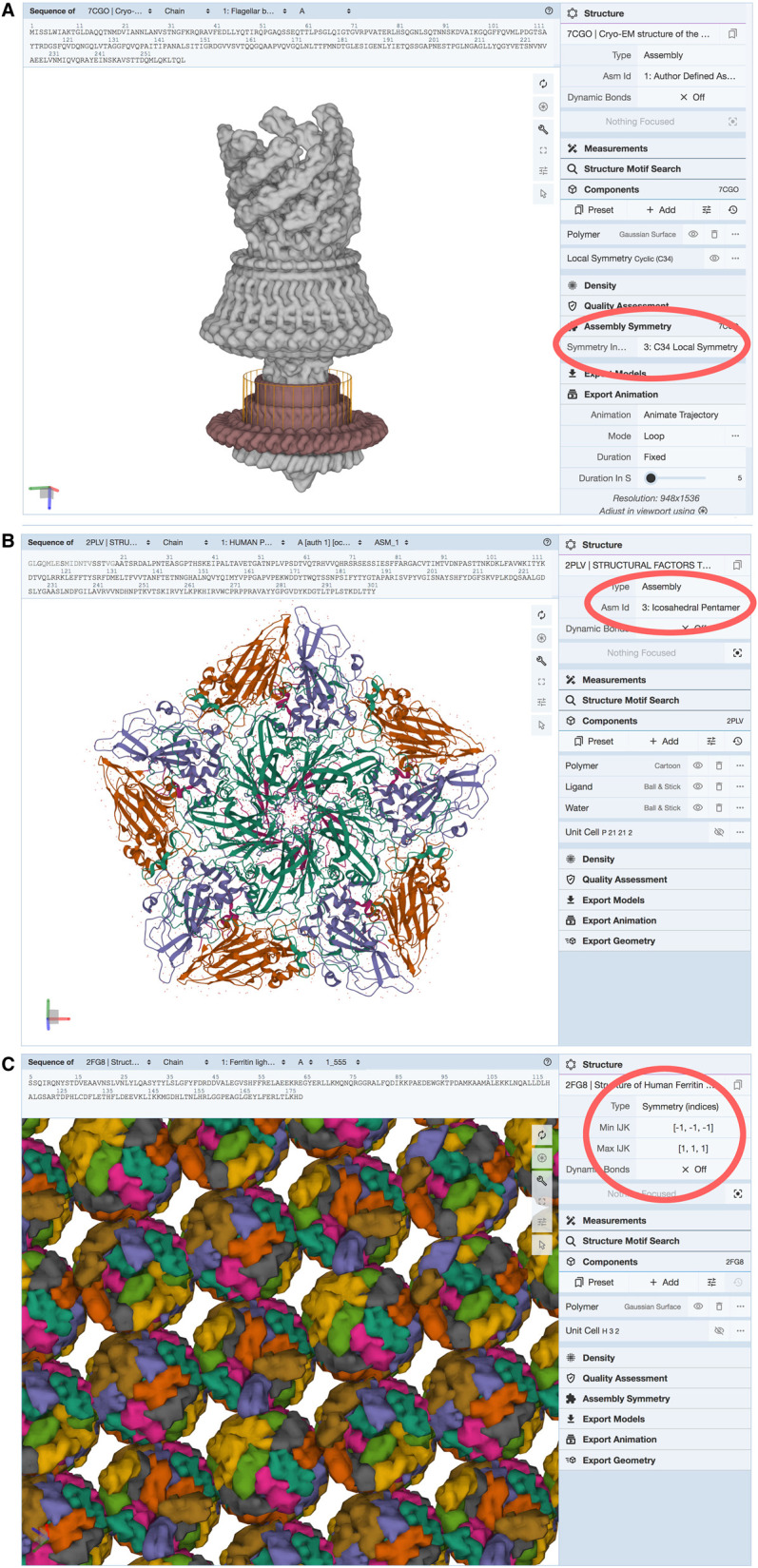
Examples of Symmetry Presets in Mol*. (**A**) The Mol* ‘Assembly Symmetry' option displays symmetry elements and a polygon representing the symmetry of the assembly. A ring with C34 local symmetry is highlighted here in a structure from a bacterial flagellar motor (PDB id 7cgo [52]). Options in the ‘Structure' panel allow display of assemblies, asymmetric units, or crystallographic lattices. Shown here are (**B**) the ‘Icosahedral Pentamer’ assembly intermediate for poliovirus (PDB id 2plv [53]) and (**C**) the ‘Symmetry (indices)' view of ferritin packing within the crystallographic lattice (PDB id 2fg8 [54]).

RCSB.org provides interactive visualization of each structure using Mol*, an advanced, open-source, web-based visualization tool designed to address the current challenges of increasing size and complexity of biostructure data. Mol* includes several tools for visualizing symmetry (Figure 6). First, the ‘Assembly Symmetry’ preset option generates a view that highlights point group and helical symmetry. This view includes symmetry axes with traditional rotation order symbols and a bounding polygon with the same symmetry, which is particularly useful in cases with complex local symmetry, as seen in Figure 6A. Second, several options in the ‘Structure’ panel allow easy display of the *asymmetric unit*, *biological assembly*, or packing of molecules within the crystal lattice. For example, in PDB structures of icosahedral virus particles, in addition to the complete icosahedral symmetry, sub-assemblies such as the icosahedral asymmetric unit, icosahedral pentamer, and where appropriate the crystal asymmetric unit can also be displayed (Figure 6B). For crystallographic structures, the ‘Structure’ panel also has options for exploring the packing of assemblies within the crystal lattice (Figure 6C).

The RCSB PDB website provides full documentation to explain use of these symmetry-related tools for students, educators, and other interested users. Documentation has been authored and updated based on user input, both through periodic surveys and feedback from the website help functionality. Documentation helps users identify tools on the website, guides them through methods to explore the type(s) of symmetry in an assembly, explains how to visualize and analyze them, and finally presents how to use the search and browse tools to find other examples of similar assemblies in the PDB archive. PDB-101, the RCSB PDB outreach and education web portal (pdb101.rcsb.org, [36]), also provides several user-friendly materials to help new users get started. A dedicated page explaining biological assemblies is available in the ‘Guide to Understanding PDB Data’, together with related introductory materials on biomolecules and how their structures are determined. PDB-101 also provides several educational materials related to symmetry, including a poster and paper-folding activity on viral quasisymmetry, paper models of icosahedral viruses, and illustrations of packing within protein crystal lattices.

## Conclusions

RCSB PDB strives to provide nimble mechanisms for accessing, visualizing, and exploring the PDB archive of atomic-level 3D biostructures. Tools presented herein are focused on functional symmetry that can readily display and support the exploration of global, local, and pseudo symmetries to help generate hypotheses regarding the functional significance of these assemblies. Analogous tools are available for applications to computer-aided drug discovery (reviewed in [37]), protein fold prediction (reviewed in [38]), and all manner of other topics that are being explored by the structural biology community. The PDB archive is growing by more than 12 000 structures per year, so these tools have been built with extensibility in mind, to ensure that newly deposited structures are accessible, and to facilitate the development of new tools that address new and evolving needs of the community.

## Summary

- Structural biologists have revealed that biomolecules exploit symmetry to achieve a wide variety of functions.
- The Protein Data Bank (PDB) is the single global repository of 3D biostructures and includes many examples of functional symmetry of biomolecules.
- The RCSB PDB website (RCSB.org) provides user-friendly tools for finding and visualizing biostructures and understanding the role of symmetry in their function.

## Abbreviations

API: application programming interface
PDBe: Protein Data Bank in Europe
PDBj: Protein Data Bank Japan
RCSB: Research Collaboratory for Structural Bioinformatics
RMSDs: root-mean-square-deviations
